# The Zoonotic *Angiostrongylus cantonensis* and the Veterinary Parasite *Aelurostrongylus abstrusus* Infecting Terrestrial Gastropods from Urban Areas of Macapá, Brazilian Amazon Region

**DOI:** 10.3390/pathogens13030255

**Published:** 2024-03-15

**Authors:** Tatiane Alves Barbosa, Silvana Carvalho Thiengo, Monica Ammon Fernandez, Jucicleide Ramos-de-Souza, Suzete Rodrigues Gomes

**Affiliations:** 1 Laboratório de Malacologia, Instituto Oswaldo Cruz, Fundação Oswaldo Cruz, Av. Brasil, 4365, Rio de Janeiro 21040-900, Brazil; endemias.dvsmcp@gmail.com (T.A.B.); sthiengo@ioc.fiocruz.br (S.C.T.); ammon@ioc.fiocruz.br (M.A.F.); suzete.gomes@ioc.fiocruz.br (S.R.G.); 2 Programa de Pós-Graduação Stricto Sensu em Vigilância e Controle de Vetores—PPG-VCV, IOC/Fiocruz, Av. Brasil 4365, Rio de Janeiro 21040-900, Brazil; 3 Departamento de Vigilância Ambiental, Divisão de Vigilância e Controle de Vetores, Secretaria Municipal de Vigilância em Saúde de Macapá, Amapá 68906-849, Brazil

**Keywords:** *Achatina fulica*, slugs, snails, zoonotic helminths, eosinophilic meningitis, *Aelurostrongylus abstrusus*

## Abstract

Metastrongyloidea includes nematodes that parasitize mammals, mainly infecting their respiratory and cardiovascular systems, and are responsible for emerging zoonosis in the world. Terrestrial mollusks are their main intermediate hosts, with few exceptions. Here we present the results of a malacological survey to know the distribution of *Angiostrongylus cantonensis* in Macapá, Amapá, in the Brazilian Amazon region, after the report of a case of eosinophilic meningitis in 2018. Mollusks were collected in 45 neighborhoods between March 2019 and February 2020. They were identified, parasitologically analyzed, and their nematodes parasites were identified based on the morphology and MT-CO1 sequencing. Infections of An. cantonensis were observed in *Achatina fulica*, *Sarasinula linguaeformis* and *Subulina octona*. These are the first records of the natural infection of the last two species by *An. cantonensis* in the Brazilian Amazon region. The angiostrongylid *Aelurostrongylus abstrusus*, which parasitizes cats, was also detected parasitizing *A. fulica* and *Diplosolenodes occidentalis*. This is also the first record of the slug *D. occidentalis* infected by *Ae. abstrusus*. The highest infection rates were recorded in neighborhoods where the environment conditions favor the proliferation of both mollusks and rodents. The results demonstrate the ample distribution of *An. cantonensis* in Macapá and the need for surveillance and mollusk vector control in Brazil and other countries.

## 1. Introduction

The superfamily Metastrongyloidea includes nematode species that parasitize primarily mammals, by infecting their respiratory and cardiovascular systems, although a few species are neurotropic [1]. Most metastrongyloid species, specifically from the family Angiostrongylidae, use terrestrial gastropods as intermediate hosts, although some are known to parasitize freshwater gastropods [2,3]. In addition to wild animals, some angiostrongylid species parasitize humans, and domestic and synanthropic animals. The most important of these angiostrongylid in public health terms is *Angiostrongylus cantonensis* (Chen, 1935), which causes cerebral angiostrongyliasis, which is also known as Eosinophilic Meningitis (EM). A second species of the same genus present in Brazil, *Angiostrongylus costaricensis* Morera and Céspedes, 1971, causes Abdominal Angiostrongyliasis (AA) [4,5,6,7].

While EM is naturally endemic to Southeast Asia and some Pacific islands, it is currently found in many regions around the world, such as the Americas, including Brazil [8,9,10,11,12,13]. Up to now, approximately 40 cases of EM have been recorded in Brazil, with the most recent case coming from the northern state of Amapá (AP) in 2020, in which the giant African land snail, *Achatina fulica* Bowdich, 1822, was identified as the transmission agent [12,14,15].

The life cycle of *An. cantonensis* involves rats as definitive hosts and various species of gastropods as intermediate hosts. Humans are accidentally infected in most cases through the ingestion of mollusks or infected paratenic hosts, as well as the consumption of foodstuffs contaminated with infectious third stage larvae (L_3_) of the nematode, which are found in the mucus of the mollusks [16,17,18]. In Brazil, *Rattus rattus* (Linnaeus, 1758) and *Rattus norvegicus* (Berkenhout, 1796) have been found with natural infection of *An. cantonensis* [3,19,20,21].

Mollusks are infected through the ingestion of first stage larvae (L_1_) released in the feces of rodents or by the active penetration of the tegument of the mollusk by the larvae. Two molts occur in the mollusk tissue (L_2_ and L_3_). A number of different species of mollusk are known to be infected naturally by *An. cantonensis*, which contributes to the persistence of the natural cycle of this parasite, and its dispersal in the environment: *Sarasinula marginata* (Semper, 1885), *Bradybaena similaris* (Férussac, 1821)*, Subulina octona* (Bruguiére, 1789), *Pomacea lineata* (Spix in Wagner, 1827) and other *Pomacea* species. [2,8,11,13,18,22].

Another genus of concern in Angiostrongylidae, is *Aelurostrongylus abstrusus* (Railliet, 1898), which parasitizes the respiratory tract of both domestic and wild felids [23,24,25] that are infected through the ingestion of parasitized mollusks or paratenic hosts [26,27]. The first stage larvae go up into the trachea, where they are swallowed and excreted in the feces [25,26]. The larvae penetrate through the mollusk tissue before developing to the third larval stage. In Brazil, *A. fulica* is the mollusk most frequently associated with *Ae. abstrusus*, although infection of the native slug *Latipes erinaceus* (Colosi, 1921) has been recorded in Rio de Janeiro [3,25].

Given this scenario of public health concern and the recent report of a case of EM in Macapá [14], the present study aimed to expand the investigation of the occurrence and distribution of *An. cantonensis* and other angiostrongylid nematodes in terrestrial mollusks in this municipality. Therefore, the main goal was to know the distribution of the nematodes that affect the health of both humans and animals and their intermediate hosts in Macapá, thus contributing to guiding surveillance and control of snail-borne parasitic diseases.

## 2. Materials and Methods

### 2.1. Study Design

The terrestrial mollusks were collected from 45 neighborhoods of Macapá over a 14-month period between March 2019 and February 2020. The collecting points included vacant lots, and public and residential gardens, in which large amounts of debris or decomposing organic material were present, given that these are favorable environments for the occurrence of terrestrial mollusks and rodents. The specimens were collected manually during active searches of each site, which lasted 10–30 min. Each point was georeferenced with a handheld Garmin 64s GPS, for plotting in ArcGIS 10.4.1.

In the laboratory, the mollusks were kept alive until the parasitological analysis. Specimens from each point were fixed for taxonomic identification based on the diagnosis of their morphology (shell and anatomy of reproductive system), supported by the relevant literature [28,29,30,31,32,33,34,35]. These specimens were deposited in the Mollusk Collection of the Oswaldo Cruz Institute (CMIOC/Fiocruz) in Rio de Janeiro.

### 2.2. Parasitological Analysis

A total of 306 mollusk specimens were analyzed individually for the presence of nematode parasites through the artificial digestion of their tissue [36,37] for releasing of the nematode larvae. These larvae were initially identified based on their morphology under a stereomicroscope (100×) and optical microscope (40×), according to Ash [38], Thiengo et al. [39], and Rodrigues et al. [25]. The larvae with diagnostic characteristics of the Metastrongyloidea were separated for the sequencing of the mitochondrial cytochrome *c* oxidase subunit I gene (COI). The larvae of *Ae. abstrusus* were identified only by their morphology, based on the presence of a rounded, button-like structure in the terminal portion of the tail.

### 2.3. Molecular Analysis

For each sample identified as *Angiostrongylus* based on the morphological criteria, 10 larvae were transferred to micro-centrifuge tubes and frozen at −18 °C in 30 μL PBS (Phosphate-Buffered Saline). The genomic DNA was then isolated by thermal shock using liquid nitrogen, according to the Standard Operating Procedure (SOP) used for this technique in the Brazilian National Reference Laboratory for Schistosomiasis and Malacology (LRNEM-IOC), where the MT-CO1 was amplified [40]. The Polymerase Chain Reaction (PCR) was run in a final volume of 50 μL containing 23.90 μL of ultra-pure water, 11 μL of 10% trehalose, 5.5 μL of 10× PCR reaction buffer, 4.4 μL of 2.5 mM dNTPs, 2.75 μL of 50 mM MgCl_2_, 1.1 μL of each primer (forward and reverse) (0.2 μM of Nem_F3 and Nem_R3, modified from Prosser et al. [40], and 0.25 μL of recombinant Taq DNA polymerase (Thermo Fisher Scientific, Waltham, MA, USA). A total of 5 μL of the DNA sample was added to the mixture, to produce a final reaction volume of 55 μL. The PCR products were purified using the Illustra GFX PCR DNA and Gel Band Purification kit (GE Healthcare, Little Chalfont, UK), following the manufacturer’s protocol. The purified products were sequenced bidirectionally using the BigDye Terminator v3.1 Cycle Sequencing kit (Applied Biosystems, Waltham, MA, USA), according to the maker’s instructions. The samples were sequenced in an ABI 3730 DNA analyzer (Applied Biosystems) installed at the DNA Sequencing Platform of the Oswaldo Cruz Institute (PDTIS/FIOCRUZ) in the RPT01A–DNA Sequencing subunit.

The chromatograms of the amplified sequences were assembled into contigs, analyzed and edited in Geneious Prime 2023.02.1 (http://www.geneious.com, accessed on 30 December 2023). This sequence was used to search GenBank (www.ncbi.nlm.nih.gov/genbank, accessed on 30 December 2023) for similar MT-CO1 sequences, using the BLAST (Basic Local Alignment Search Tool) in the BLASTn algorithm [41]. Sequences of *An. cantonensis* obtained from GenBank were used for the phylogenetic analyses, with four taxa of the genus *Angiostrongylus* being used as the outgroup, and one taxon of the genus *Aelurostrongylus*.

The MT-CO1 sequences were aligned using the Muscle tool, which was implemented in Geneious R9 [42] and the resulting matrix was edited to eliminate poorly aligned extremities and converted to the Nex format in Mesquite for the construction of the phylogenetic tree, version 3.51 [43]. The Markov chains were configured in the command block to be sampled at every 100 generations (sampleFreq = 100) in a total run of 10 million generations (ngen = 10,000,000). The posterior probabilities were calculated from the residuals, and a consensus sequence was generated based on the 50% majority rule.

Analyses of Bayesian Inference (BI) were run in MrBayes version 3.2.7 [44], using the GTR+I+G evolutionary model. The Bayesian analysis was run in the CIPRES Science Gateway V. 3.3 (https://www.phylo.org/, accessed on 30 December 2023) [45].

## 3. Results

**Figure 1:**
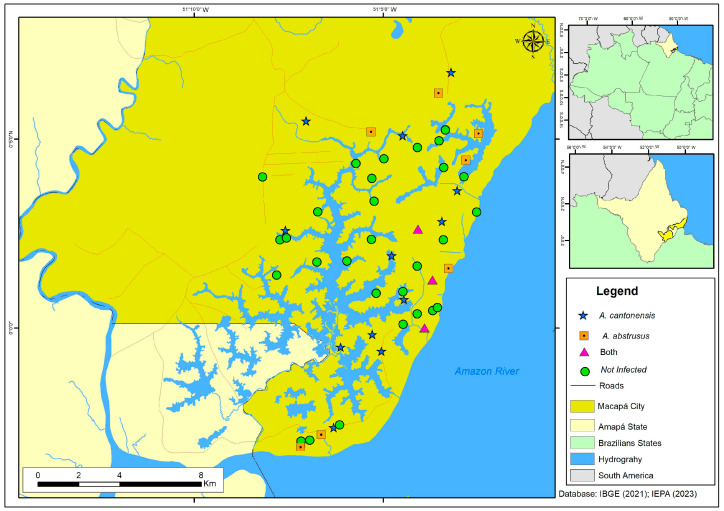
Study area in the municipality of Macapá, in northern Brazil, indicating the points from which mollusks were collected. Blue star: sites where the mollusks were positive for *An. cantonensis*; orange square: sites where mollusks were positive for *Ae. abstrusus*; green circle: site where the mollusks were not positive for Metastrongyloidea larvae; pink triangle: sites where both species were found, *An. cantonensis* and *Ae. abstrusus*.

In all five species of terrestrial mollusks were collected: *A. fulica* (*n* = 159), *Bulimulus tenuissimus* (d’Orbigny, 1835) (*n* = 35), *Subulina octona* (*n* = 19), and the slugs *Diplosolenodes occidentalis* (Guilding, 1825) (*n* = 29) and *Sarasinula linguaeformis* (Semper, 1885) (*n* = 64). The parasitological examination of 306 of the specimens collected revealed that 163 were parasitized by nematodes. Of these, 59 specimens presented angiostrongylid larvae from 29 of the 45 neighborhoods of Macapá (Figure 1 and Table 1). Except for *B. tenuissimus*, at least one specimen of each mollusk species was infected by angiostrongylid.

The localities of Beirol and Marabaixo III showed greater epidemiological importance (seven and six snails parasitized by Metastrongyloidea, respectively), unlike the other three neighborhoods (Buritizal, Jardim Equatorial, Jardim Marco Zero, Novo Buritizal, Parque dos Buritis, and São Lázaro). Mollusks infected with An. cantonensis and Ae. abstrusus were collected at 15 and 11 localities, respectively (Table 1). Both nematode species were recorded in three neighborhoods (Beirol, Jardim Felicidade, and Jesus de Nazaré).

**Figure 2:**
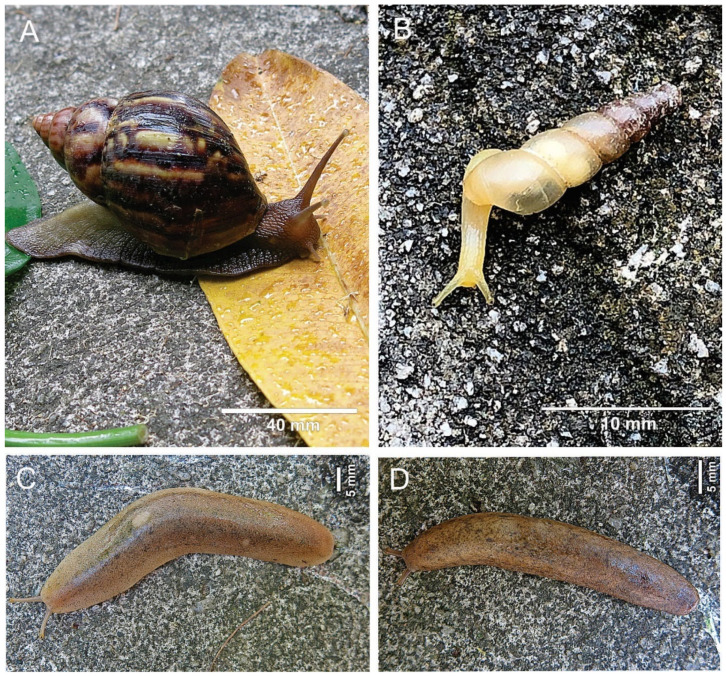
Photos of live specimens of the species found parasitized with Metastrongyloidea larvae in Macapá, Macapá, Brazilian Amazon Region: (**A**)—*Achatina fulica*, (**B**)—*Subulina octona*, (**C**)—*Diplosolenodes occidentalis*, (**D**)—*Sarasinula linguaeformis*.

**Figure 3:**
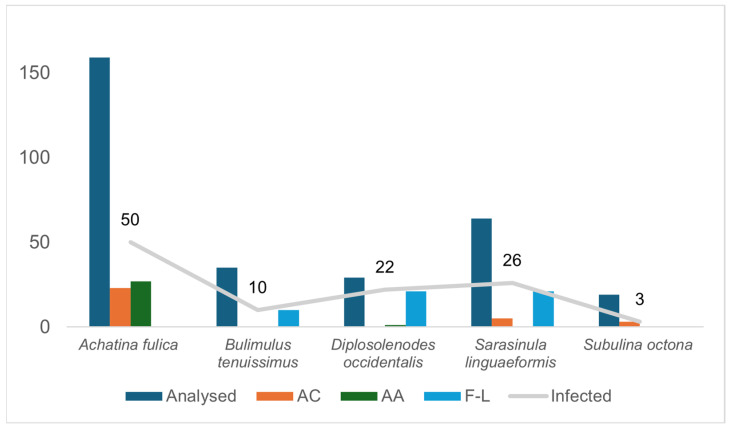
Results of the parasitological analysis of gastropods collected in the municipality of Macapá, in northern Brazil. AC: gastropods infected with *An. cantonensis*, AA: gastropods infected with *Ae. abstrusus*, F-L: free-living nematodes. Grey line indicated the total of infected specimens.

Infections of *An. cantonensis* were observed in *A. fulica* (23 specimens: 14.11% of the total of this species analyzed parasitologically), *S. linguaeformis* (five specimens: 7.81%), and *S. octona* (three specimens:15.78%); *Ae. abstrusus* was detected in only two species: *A. fulica* (27 specimens: 16.98%) and *D. occidentalis* (one: 3.44%) (Figure 2 and Figure 3). The invasive exotic species *A. fulica* was the only mollusk parasitized by both nematodes (Table 1).

Of the 163 infected mollusks, 36.19% were parasitized with *An. cantonensis* and *Ae. abstrusus* larvae, and the hosts were predominantly *A. fulica* (51.96% of the total snails collected in the area). Considering the 306 specimens obtained in Macapá, by species collected, the indices of infection by Metastrongyloidea (*An. cantonensis, Ae. abstrusus* and larvae not identified due to the low parasitic load) and other nematodes can be observed in Table 2. The terrestrial snail *B. tenuissimus* was represented by 35 specimens (11.43% of mollusks obtained in the area), with snails infected by only non-harmful nematodes (ten snails: 28.57% of the specimens of this species obtained in the area).

**Figure 4:**
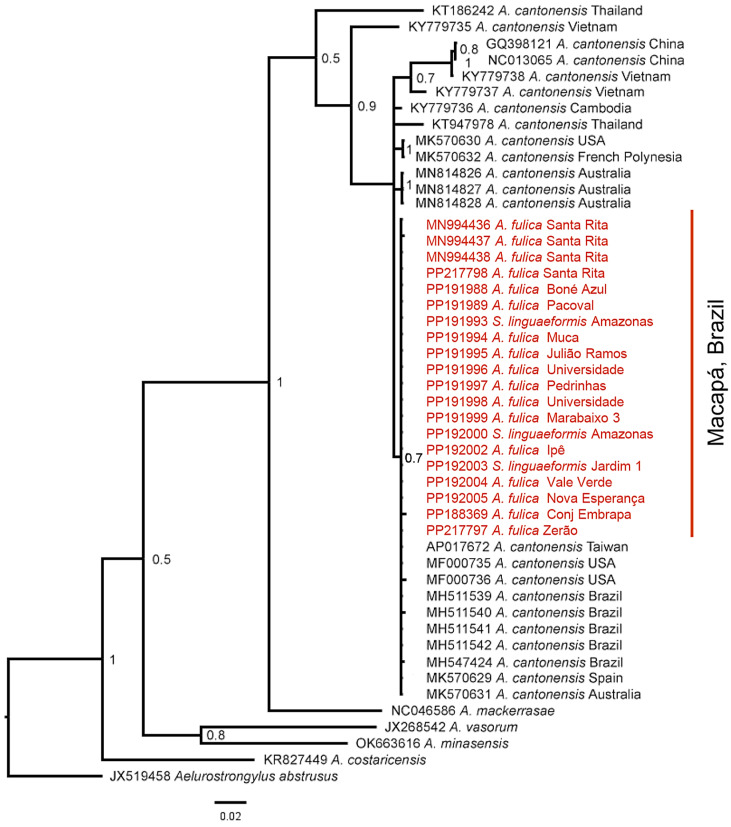
Phylogenetic reconstruction based on the partial sequences of the mitochondrial cytochrome *c* oxidase subunit 1 (CO1) gene using the Bayesian Inference (BI) approach. The 50% majority consensus tree obtained using the GTR + I + G model is shown. The numbers at the branch nodes are the Bayesian posterior probabilities of the 48 sequences, which include the 20 obtained from the present study, which are shown in pink, with their respective hosts in blue. *Aelurostrongylus abstrusus* was included as the outgroup.

The sequencing of the CO1 gene generated two good sequences (forward and reverse), resulting in 700 base pairs for each *An. cantonensis* sample. The present study generated 18 new sequences of *An. cantonensis* obtained from 14 specimens of *A. fulica*, three *S. linguaeformis*, and one *S. octona*. All the new sequences have been deposited in GenBank. In the phylogenetic analysis (Figure 4) we included 17 of these sequences (see Table 3) and three sequences from Amapá, previously published by Barbosa et al. [14] (GenBank accession numbers MN994436, MN994437, and MN994438). Some of the sequences were abnormally short and were not considered to be adequate for analysis. All the sequences recovered in the present study were of the same haplotype and were 99.5–100% similar to the *An. cantonensis* sequences deposited in GenBank.

The sequences obtained in the present study formed a distinct clade (BPP = 75%), which clustered with the *An. cantonensis* sequences from Australia, Brazil, Spain, and Taiwan. All the *An. cantonensis* sequences form a monophyletic group, albeit with low support, i.e., BPP = 53% (Figure 4). Monophyletic groups were observed among the different species of the same genus, such as *An. cantonensis* (BPP = 50%) and *Angiostrongylus mackerrasae* (Bhaibulaya, 1968) (BPP = 100%). The *An. cantonensis* sequence obtained in the present study from *S. octona* was excluded due to its abnormally short length.

## 4. Discussion

Our results confirm and expand on the participation of the terrestrial mollusks in the maintenance of the life cycle of important metatrongyloids in Brazil, including *An. cantonensis* and *Ae. abstrusus.* Although Barbosa et al. [14] reported these nematodes in Macapá, the malacological investigation indicated only *A. fulica* acting in the transmission in the Santa Rita neighborhood, where the case of EM was reported in the municipality in 2018. Barbosa et al. [14] recorded *A. fulica* infected with *Ae. abstrusus* in the Santa Rita neighborhood of Macapá.

Both *An. cantonensis* and *Ae. abstrusus* have also been recorded in other regions of Brazil [3,21], as well as in other countries [2]. Carvalho et al. [22] reported the infection of a number of different mollusk species by *An. cantonensis* in areas adjacent to ports in different Brazilian states. Bechara et al. [56] also reported the occurrence of *A. fulica* and infected definitive hosts in anthropogenic areas of the municipality of Rio de Janeiro, in southeastern Brazil. Infestations of *A. fulica* are typically found on vacant lots, where refuse and decomposing organic material tends to accumulate [21,57,58], as observed at many of the sites in Macapá.

The *An. cantonensis* larvae obtained from the mollusks collected in Macapá and analyzed using CO1 sequences were 99.5~100% similar to the catalogued *An. cantonensis* sequences recovered in the BLAST search. In the phylogenetic tree obtained here, the *An. cantonensis* samples were closest to the other sequences obtained from Brazil, such as those identified in the state of Sergipe [51]. But they also clustered with sequences from Australia, Spain, and Taiwan, which indicates a closer relationship with *An. cantonensis* from these regions. A similar relationship between Brazilian *An. cantonensis* and populations from Asian countries was found by Monte et al. [59], who observed that the Brazilian sequences were close to those from Japan, China, and Thailand. These findings may reflect the proximity of ports at which ships carrying definitive (rodents) or intermediate hosts (mollusks) infected with *An. cantonensis* have docked [26,59,60,61]. The present study is the first to analyze samples of *An. cantonensis* from the Brazilian state of Amapá. Given the rapid dispersal of the snail *A. fulica* in Brazil and the fact that *An. cantonensis* has been found infecting these snails in 14 Brazilian states up to now [3,12,14,21,51,61,62], phylogenetic studies of this parasite may contribute to the understanding of the dynamics of its introduction to and dispersal in the country. *An. cantonensis* was also reported from other Brazilian states, i.e., Rio de Janeiro, Espírito Santo, Goiás, Mato Grosso, São Paulo, Sergipe, Minas Gerais [27,39], and Amazonas [63]. 

Rodrigues et al. [25] recently also found a strong association between this nematode and *A. fulica*, based on the parasitological analysis of mollusks collected from urban areas in 46 of the 92 municipalities of the state of Rio de Janeiro. These authors also recorded *Ae. abstrusus* in the veronicellid slug *Latipes erinaceus*. Penagos-Tabares et al. [23,24] also reported the infection of *Achatina fulica* by *Ae. abstrusus* in Colombia.

The present study also identified new associations between these nematodes and terrestrial mollusks in Macapá, specifically, the natural infection of the veronicellid slugs *S. linguaeformis* and *D. occidentalis* by *An. cantonensis* and *Ae. abstrusus*, respectively. Robinson et al. [64] reported that *D. occidentalis* occurs in the Lesser Antilles, Central America, and northern South America. The present study reports the first record of the species in Brazil as well as of its infection by *An. cantonensis*. By contrast, *An. cantonensis* has already been recorded infecting *S. linguaeformis* in the Brazilian states of São Paulo, Bahia, Espírito Santo, Pernambuco, and Pará [12,22].

A previous malacological research study revealed 21 species of exotic and native terrestrial molluscs to the city of Macapá. In the present study, an epidemiological investigation revealed the association of nematodes with some of these species of terrestrial mollusks [65]. *Achatina fulica* was the most common and widespread gastropod species in the study area. It was also the mollusk most infected by the nematodes, as well as the only species infected by both *An. cantonensis* and *Ae. abstrusus*. These findings further reinforce the importance, not only for the dispersal of *An. cantonensis*, but also the maintenance of its life cycle, in a number of different countries around the world [2,3,17,22].

While *B*. *tenuissimus* was not found to be infected naturally by metastrongyloid larvae in Macapá, Ramos-de-Souza et al. [51] recorded the association of *An. cantonensis* and this species in the Brazilian state of Sergipe. This mollusk is amply distributed in Brazil, where it has been recorded in eight states: Pará, Maranhão, Pernambuco, Bahia, Mato Grosso, Espírito Santo, Rio de Janeiro, and São Paulo [30]. In an experimental study designed to evaluate the potential of *B. tenuissimus* as an intermediate host, Martins et al. [66] found that its susceptibility to infection by *An. cantonensis* was 17.25%.

The highest nematode infection rates were recorded in the Beirol, Marabaixo III, and Pedrinhas neighborhoods, where the social and environmental conditions favor the proliferation of both mollusks and rodents. In northern Brazil, infected *Achatina fulica* had also been collected in the municipality of Barcelos, in the state of Amazonas, as well as in the city of Belém, in Pará state, together with infected specimens of *R. rattus* and *R. norvegicus* [67].

Exotic species often have a disproportionate impact on the environment, given their ecological plasticity, lack of natural predators, and their potential for the transfer of parasites to the native fauna, a process known as “spillover” [2]. The results of the present study indicate that the giant African land snail *Achatina fulica* is a typical example of this problem, given its ample dispersal throughout all the states in Brazil, as well as the Federal District, where it is abundant in many urban areas, especially where refuse and debris accumulate, which favors the presence of rodents and the completion of the life cycle of *An. cantonensis* and other zoonotic nematodes [3,12].

Webster et al. [68] highlighted the One Health concept, which associates the health of human populations with that of both animals and the environment, with a focus on the transmission of zoonotic diseases. Zinsstag et al. [69] also reinforced the importance of the One Health concept, which they associate with the term One Medicine, which emphasizes the need for the integration and convergence of the health of all the species and the ecosystems they inhabit. Woldehanna and Zimicki [70] also pointed out the importance of identifying the relationship between human behavior and the probable infection routes, to ensure the development of effective preventive measures. In particular, the social dynamic determines the level of possible interactions with animals, as well as the intensity of these interactions and, in turn, the potential for exposure to pathogens.

## 5. Conclusions

The present study demonstrated the ample distribution of *An. cantonensis* in the municipality of Macapá and the maintenance of its life cycle through the presence of different species of terrestrial mollusk, besides *A. fulica.* It also provides the first record of the occurrence of the slug *Diplosolenodes occidentalis* infected by *Ae. abstrusus* in Brazil, as well as the first records of the natural infection of *Subulina octona* and *Sarasinula linguaeformis* by *An. cantonensis* in the Brazilian Amazon region.

The finding of different species of mollusks infected with parasitic nematodes highlights the importance of such studies for public and veterinary health. We also emphasize the need for the implementation of measures to improve the health of the local population, in particular through health education, and the surveillance and control of the mollusk intermediate hosts. These measures are fundamental to the prevention of the transmission of neglected zoonotic diseases, in particular EM, in urban areas where infected mollusks are relatively abundant.

## Figures and Tables

**Table 1:** The neighborhoods of the municipality of Macapá in which mollusk specimens infected by angiostrongylid nematode larvae were collected during the present study. The values indicate the number of individuals infected by each type of nematode in each neighborhood. AA = *Aelurostrongylus abstrusus*, AC = *Angiostrongylus cantonensis*.

	*Achatina fulica*		*Subulina octona*	*Diplosolenodes occidentalis*	*Sarasinula linguaeformis*
Neighborhoods	AA	AC	AC	AA	AC
Amazonas					2
Beirol	1	4	2		
Boné Azul		2			
Embrapa		1			
Fazendinha	4				
Ipê	1				
Jardim Felicidade I	1			1	1
Jardim Felicidade II	4				
Jesus de Nazaré	1	1			
Julião Ramos		1			
Marabaixo III		3	1		2
Muca		1			
Murici	4				
Nova Esperança		2			
Pacoval		1			
Palmeiras	2				
Pedrinhas		3			
Renascer I	3				
Santa Inês	1				
São José	5				
Universidade		2			
Vale Verde		1			
Zerão		1			

**Table 2:** Mollusk species infected by nematode larvae collected in Macapá. The values indicate the number of individuals infected (*n*) by each species of nematode and the percentage of infection considering the total number of parasitized mollusks.

Species	Collected (*n*)	Parasitized (*n*)	Metastrongyloidea, Angiostrongylidae			Other Nematodes (*n*)
			*Angiostrongylus* *cantonensis* Only (*n*)	*Aelurostrongylus* *abstrusus* Only (*n*)	Not Identificated	
*A. fulica*	159	108	23 (14.11%)	27 (16.56%)	10 (6.13%)	48 (29.45%)
*B. tenuissimus*	35	10	-	-		10 (6.13%)
*D. occidentalis*	29	21	-	1 (0.61%)		20 (12.27%)
*S. linguaeformis*	64	21	5 (3.06%)	-	4 (2.45%)	12 (7.36%)
*S. octona*	19	3	3 (1.84%)	-		-
Total	306	163				

**Table 3:** The nematode CO1 sequences included in the phylogenetic analysis of the present study, with their respective taxonomic classification, country, host, accession number, and reference.

Species	Geographic Location	Hosts	GenBank Acession Number	References
*Angiostrongylus cantonensis*	China	*Rattus norvegicus*	GQ398121	Lv et al. [46]
	China	*Rattus norvegicus*	NC013065	Lv et al. [46]
	Tailand	*Mus musculus*	KT947978	Yong et al. [47]
	USA	*Rattus exulans*	MK570630	Cervená et al. [48]
	French Polynesia	*Rattus exulans*	MK570632	Cervená et al. [48]
	Australia	*Rattus fuscipes*	MN814826	Valentyne et al. [49]
	Australia	*Rattus rattus*	MN814827	Valentyne et al. [49]
	Australia	*Rattus rattus*	MN814828	Valentyne et al. [49]
	Brazil	*Achatina fulica*	MN994436	Barbosa et al. [14]
	Brazil	*Achatina fulica*	MN994438	Barbosa et al. [14]
	Brazil	*Achatina fulica*	MN994437	Barbosa et al. [14]
	Brazil	*Achatina fulica*	PP188369	Present study
	Brazil	*Achatina fulica*	PP191988	Present study
	Brazil	*Achatina fulica*	PP191989	Present study
	Brazil	*S. linguaeformis*	PP191993	Present study
	Brazil	*Achatina fulica*	PP191994	Present study
	Brazil	*Achatina fulica*	PP191995	Present study
	Brazil	*Achatina fulica*	PP191996	Present study
	Brazil	*Achatina fulica*	PP191997	Present study
	Brazil	*Achatina fulica*	PP191998	Present study
	Brazil	*Achatina fulica*	PP191999	Present study
	Brazil	*S. linguaeformis*	PP192000	Present study
	Brazil	*Achatina fulica*	PP192002	Present study
	Brazil	*S. linguaeformis*	PP192003	Present study
	Brazil	*Achatina fulica*	PP192004	Present study
	Brazil	*Achatina fulica*	PP192005	Present study
	Brazil	*Achatina fulica*	PP217797	Present study
	Brazil	*Achatina fulica*	PP217798	Present study
	Taiwan	*-*	AP017672	Unpublish
	USA	*Didelphis virginiana*	MF000735	Dalton et al. [50]
	USA	*Didelphis virginiana*	MF000736	Dalton et al. [50]
	Brazil	*Achatina fulica*	MH511539	Ramos-de-Souza et al. [51]
	Brazil	*Achatina fulica*	MH511541	Ramos-de-Souza et al. [51]
	Brazil	*Cyclodontina fasciata*	MH511542	Ramos-de-Souza et al. [51]
	Brazil	*Bulimulus tenuissimus*	MH547424	Ramos-de-Souza et al. [51]
	Spain	*Rattus rattus*	MK570629	Cervená et al. [48]
	Australia	*Rattus rattus*	MK570631	Cervená et al. [48]
	Tailand	-	KT186242	Yong et al. [52]
	Cambodja	*Pomacea* sp.	KY779735	Lv et al. [53]
	Cambodja	*Pomacea* sp.	KY779736	Lv et al. [53]
	Vietnam	*Pomacea* sp.	KY779737	Lv et al. [53]
	Vietnam	*Pomacea* sp.	KY779738	Lv et al. [53]
*Angiostrongylus mackerrasae*	Australia	*Rattus fuscipes*	MN814821	Valentyne et al. [49]
	Australia	*Rattus fuscipes*	MN814822	Valentyne et al. [49]
	Australia	*Rattus fuscipes*	MN793157	Valentyne et al. [49]
	Australia	*Rattus fuscipes*	NC046586	Valentyne et al. [49]
*Angiostrongylus malaysiensis*	Malaysia	*Rattus rattus diardii*	KT947979	Yong et al. [47]
	Malaysia	*Rattus rattus diardii*	NC_030332	Yong et al. [47]
*Angiostrongylus minasensis*	Brazil	*Nasua nasua*	OK663616	Almeida et al. [4]
	Brazil	*Nasua nasua*	OK663635	Almeida et al. [4]
*Angiostrongylus costaricensis*	Costa Rica	*Nasua narica*	KX378965	Santoro et al. [54]
	Costa Rica	-	AP017675	Unpublish
	Costa Rica	-	KR827449	Yong et al. [55]

## Data Availability

Mollusks samples were deposited in the Mollusk Collection of the Oswaldo Cruz Institute (CMIOC/Fiocruz) in Rio de Janeiro. The database of this collection can be acessed in the following address specieslink.net. The sequences of *An. cantonensis* generated in this study were deposited in GenBank GenBank Overview (nih.gov).
